# Genetic susceptibility of serum cholesterol and triglyceride in Chinese Han children and adolescents

**DOI:** 10.1186/1687-9856-2013-S1-O38

**Published:** 2013-10-03

**Authors:** Wei-fen Zhu, Li Liang, Chun-lin Wang

**Affiliations:** 1 Department of Endocrinology, The Children’s Hospital of Zhejiang University School of Medicine, Hangzhou, China

## Objective

Genetic studies might provide new insights into the biological mechanisms underlying lipid metabolism and risk of Cardiovascular disease. We therefore conducted a study to identify genetic determinants of triglycerides(TG), high-density lipoprotein cholesterol (HDL-C) and non-high-density lipoprotein cholesterol (non-HDL-C).

## Methods

We investigated adiponectin receptor 1(AdipoR1) gene rs10920533 associated with non-HDL-C(case: 109 subjects with high non-HDL-C; control: 701 subjects with normal non-HDL-C); apolipoprotein A5(ApoA5) gene -1131T>C and -3A>G associated with TG(case: 245 subjects with high TG; control: 595 subjects with normal TG); glucocorticoid receptor(GR) gene rs12521436 associated with HDL-C(case: 129 subjects with low HDL-C; control: 722 subjects with normal HDL-C). Matrix-assisted laser desorption/ionization time-of-flight mass spectrometry (MALDI-TOF MS) was used for SNP genotyping.

## Results

Compared to AdipoR1 rs10920533 GG genotype, carriers of AG had lower non-HDL-C, but the differences did not reach statistical significance; after adjusted for BMI, TC, TG and LDL-C with multi-factor logistic regression, AdipoR1 rs10920533 AG genotype was not independently associated with the non-HDL-C level. APOA5 -1131CC and -3GG had higher TG compared to common allele homozygotes, respectively, but the differences did not reach statistical significance; after adjusted for age, WC, TC, HDL-C and LDL-C with multi-factor logistic regression, the risk of -1131CC genotype was found significantly increased than that of the TT genotype, the OR value of which was 2.667(95% confidence interval: 1.413-5.033) and the risk of -3GG genotype was found significantly increased than that of the AA genotype, the OR value of which was 2.561(95% confidence interval: 1.342-4.888). For GR rs12521436, compared to the homozygous for the G allele, carriers of A allele had lower levels of HDL-C, but the differences did not reach statistical significance; after adjusted for BMI, TC and TG with multi-factor logistic regression the result show that a more increased risk for lower HDL-C in subjects with the A carriers, which the crude OR for subjects with A allele was 1.937 (95% confidence interval: 1.166-3.215) relative to GG carriers.

## Conclusion

Our results demonstrate an independent risk for ApoA5 -1131T>C and -3A>G gene polymorphisms in the development of a elevated TG level. GR rs12521436 gene polymorphisms might contribute to a reduced level of HDL-C. The single nucleotide polymorphism rs10920533 in the AdipoR1 gene is not associated with non-HDL-C level.

